# Primary Caregivers Caring for a Child at End of Life in Saudi Arabia

**DOI:** 10.1089/pmr.2021.0072

**Published:** 2022-08-09

**Authors:** Shahad A. Hafez, Julia A. Snethen, Murad Taani, Emmanuel Ngui, Julie Ellis, Abdullah A. Baothman

**Affiliations:** ^1^ Nursing Program, Batterjee Medical College, Aseer, Saudi Arabia.; ^2^ Sigma Theta Tau International (STTI), Eta Nu Chapter, Milwaukee, Wisconsin, USA.; ^3^ College of Nursing, University of Wisconsin-Milwaukee, Milwaukee, Wisconsin, USA.; ^4^ School of Public Health, University of Wisconsin-Milwaukee, Milwaukee, Wisconsin, USA.; ^5^ King Saud Ben Abdulaziz University for Health Science, Riyadh, Saudi Arabia.

**Keywords:** children at end of life, end of life, end of life care, primary caregivers, Saudi Arabia

## Abstract

**Background:**

Caring for children at end of life (EOL) can be devastating for primary caregivers who are responsible for the physical, social, and emotional needs of their dying child. Limited information was found on resources in Saudi Arabia to manage the impact on primary caregivers from caring for a child receiving end of life care (EOLC).

**Purpose:**

The purpose of this study was to explore the experiences of primary caregivers caring for a child receiving EOLC within the Saudi Arabian health care system.

**Methods:**

A descriptive phenomenological study was conducted, and 24 female primary caregivers were interviewed individually. Participants were recruited from three hospitals and the surrounding community in Jeddah, Saudi Arabia. The data were collected over a period of seven weeks between August and September of 2019. Individual in-depth interviews were conducted using an 11-item investigator-developed interview guide derived from the literature on EOL. Thematic analysis was completed using transcripts from all interviews.

**Results:**

The findings suggest that primary caregivers caring for a child receiving EOLC were impacted psychologically, physically, socially, and financially. Primary caregivers expressed their heartbreak, lack of sleep, isolation, and financial challenges while caring for their child at EOL.

**Conclusions:**

Similar to what has been reported in the literature, primary caregivers caring for a child at EOL experience biopsychosocial and financial challenges. In addition, this study has implications for nursing education, practice, policy, and research regarding EOLC. Also, the findings can guide future research on EOL in Saudi Arabia and worldwide.

## Introduction

Taking care of a child at the end of life (EOL) is a challenging experience for primary caregivers.^1^ In Saudi Arabia, mothers or female caregivers are almost universally the primary caregivers for their children regardless of the type or severity of the child's illness.^2^ The primary caregiver intimately experiences their child's life-limiting illness.^3^ Facing a child's death can be traumatic, impacting the primary caregivers psychologically, physically, and socially.^3^ The goal of end of life care (EOLC) is to provide comfort to the dying individual by managing their pain and other undesired symptoms.^4^

There have always been ethical dilemmas regarding EOL decision making in Saudi Arabia.^5^ Usually, several individuals are involved in the decision-making process, which makes it more complicated. Individuals involved in the decision-making process in Saudi Arabia are the patient, family members, and health care providers.^6^ In Saudi Arabia, health care institutions follow the Ministry of Health policies, which are based on Islam laws and regulations. The idea that family members are not qualified to make decisions is based on a *Fatwa* developed in 1988. Fatwa number 12086 states that: “if three knowledgeable and trustworthy physicians agreed that the patient condition is hopeless; the life-supporting machines can be withheld or withdrawn. The family members' opinion is not included in decision making as they are unqualified to make such decisions.”^6^ This study aimed at exploring the experiences of primary caregivers caring for a child at EOL in Saudi Arabia.

## Methods

### Conceptual framework

**FIG. 1.**
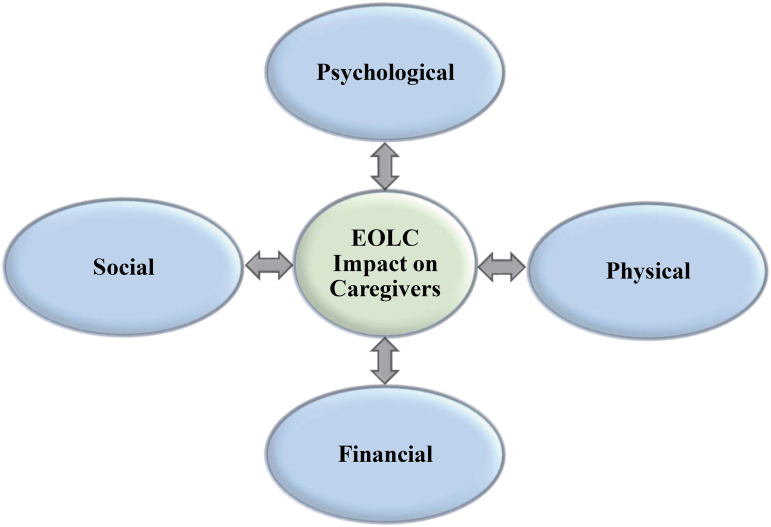
Conceptual framework.

Four main concepts emerged from the literature regarding pediatric EOLC, which were used to develop an initial conceptual framework for this study. On completion of the study, the conceptual framework was adapted to reflect the initial concepts and the current findings. The framework that emerged integrates the concepts from the literature and parental experiences caring for their child at EOL (Fig. 1).

### Design, sample, and setting

This investigation was part of a larger phenomenological study that addressed how caring for a child receiving EOLC impacts the primary caregivers' psychological, physical, social, and financial status. A purposive sample of participants were recruited for this investigation, and snowball sampling was used to further expand the participants who were recruited from the community. The inclusion criteria were: (1) Saudi Arabian female primary caregivers (mother, grandmother, aunt, sister, or stepmother) caring for a child at EOL, (2) 18 years of age or older, (3) caring for child(ren) diagnosed with a terminal illness or critical condition, (4) child(ren) between 1 day and 14 years of age, (5) and were able to give informed consent.

Participants were recruited from three urban hospitals and the surrounding communities in Jeddah in Saudi Arabia. The interviews were conducted while assuring that the child was observed and cared for during that time. IRB approval was obtained at all appropriate institutions before conducting the investigation, including the investigators' university, the Saudi Ministry of Health, and health care organizations.

### Measures

Demographic data were collected in addition to an 11-item investigator-developed interview guide. The interview guide was a template of open-ended questions derived from the EOL literature.

### Data collection

A master's prepared PhD student conducted individual in-depth interviews with 24 participants under the supervision of a senior PhD researcher. The interviews were conducted in Arabic by the investigator whose first language was Arabic. The interviews lasted between 15 minutes and an hour, depending on how much the participant wanted to share. The investigator started the interviews by asking about less sensitive topics, followed by topics that were potentially more sensitive. Following the interview, female caregivers were offered gift cards as appreciation for the time they provided to participate in the study. The data were collected over a period of seven weeks.

### Data analysis

The interviews were transcribed in Arabic and translated into English, and then back translated into Arabic. The investigator reviewed all audio recordings and transcripts for accuracy and consistency in both Arabic and English. A thematic analysis process was followed to analyze the data, using the English version of the transcripts. The research team individually coded each of the interviews. Then, the team discussed the initial coding to ensure there was an agreement regarding the coding. The investigators considered the most accurate coding for each section of the interviews until a consensus was reached. From the coded data, the emerging themes and subthemes were identified and organized into tables (Table 2).

## Results

### Participants

Caregivers included in this study were mothers (*n* = 22), a sister (*n* = 1), and a nanny (*n* = 1), ranging in age from 18 to 47 years. A majority of the participants (*n* = 20) were homemakers. Eleven participants were pre-college graduates, and six were college graduates. Four participants did not share their education level. Participants were caring for 25 children (13 females and 12 males), ranging in age from 11 days to 13 years old. A majority of the children (*n* = 18) were hospitalized, with the remaining (*n* = 7) being cared for at home (Table 1).

**Table 1.** Participants and Children Characteristics

Relationship to the child	Participant age	Child gender	Child age	Duration of illness
Mother	33	Female	5 months	5 months
Mother	30	Male	8 years	1 year and 5 months
Mother	30	Female	4 years and 6 months	4 years and 6 months
Mother	38	Male (Twins)	3 years and 7 months	3 years
Mother	41	Female	11 years	8 months
Mother	42	Female	13 years	13 years
Mother	37	Male	1 year and 7 months	1 year and 2 months
Mother	35	Female	7 months	7 months
Mother	37	Female	3 months	3 months
Mother	34	Male	11 days	11 days
Mother	42	Female	4 months	4 months
Mother	47	Male	6 years	3 years
Sitter	18	Female	3 years	2 years and 9 months
Mother	27	Male	10 years	Since birth and got complicated during the last week
Mother	35	Male	11 years	Since birth and got complicated 2 months ago
Mother	26	Male	3 years	2 years and 8 months
Mother	35	Female	9 years	2 years
Mother	35	Female	3 years	3 months
Sister	20	Female	11 years	6 months
Mother	31	Male	13 years	6 months
Mother	40	Male	4 years and 4 months	10 months
Mother	25	Female	6 years and 6 months	9 months
Mother	46	Male	2 years and 5 months	1 year
Mother	40	Male	6 years	4 months

**Table 2.** Themes

Participant	Negative psychological impact	Negative physical impact	Negative social impact	Financial challenges
1	x	x	x	
2	x		x	x
3	x	x		
4	x			
5	x	x	x	
6	x			x
7	x	x	x	x
8	x	x	x	x
9	x		x	
10	x	x	x	x
11	x	x		
12	x			x
13	x	x		
14	x	x	x	x
15	x			x
16	x			x
17	x	x	x	
18	x	x	x	x
19	x	x	x	x
20	x			x
21	x	x		x
22	x		x	x
23	x			x
24	x	x	x	x

The children's life-limiting conditions included: congenital anomalies, neurodegenerative disease, and cancer. All the hospitalized children had do not resuscitate (DNR) orders completed and included in their charts. Table 3 presents the themes, subthemes, sample quotations, and the interview questions (previously published), focused on four different areas of exploration: psychological, physical, social, and financial.^7^

**Table 3.** Themes, Subthemes, and Sample Quotations

Psychological impact	
Shock and disbelief	01: “I'm in shock! I am still in shock … I mean I am affected until today …5 months and I can't get over it”
	07: “After that shock [child was unconscious] I had, every day was a shock … but that day I couldn't help myself when I noticed that he wasn't moving his hand … there were other shocks, but not like this one!”
	12: “The doctors said cancer, I said come on people … I was in shock at first, cancer!? Not a flu? … I was surprised he has the disease [cancer] … it was a shock! My life flipped upside down”
My heart was breaking	07: “Allah bring my child back to life and kill me! I don't want anything from this world!”
	24: “I cried all the time and my heart was breaking seeing my son getting worse every day!”
How could I let go of a piece of my heart?	08: “I got used to her laugh … she knows me when she sees me, she smiles … she smiles even when she is sick at the hospital … Her father said maybe she recognizes your scent/smell”
	09: “at home I feel bitter because I am far from her … when I come to the hospital I feel that my pain gets worse when I see her like this … when I leave I don't feel comfortable … when I leave I ache because I'm leaving her here alone … every time of the day I ache differently”
Physical Impact	
When you are sad, you don't eat well	03: “I lost weight in the beginning I swear in a very sudden way”
	11: “I lost my appetite”
	13: “I don't eat or drink I just keep looking at the machine, I want her oxygen to be 100, I want it to get better!”
I sleep, but not sleep	08: “Sleep, I can't sleep I just keep thinking how is she [child] going to walk?”
	22: “It was hard to sleep, I couldn't sleep! [when at the hospital]”
Social impact	
Isolated themselves from the entire world	09: “Aaaah most of the time at home … almost isolated”
	24: “I wasn't treating people the way I used to … I didn't want to talk to people, didn't want to go out … I felt like I just want to be alone … it's hard”
Can't go out and leave the child alone	05: “I can't go everywhere, I had to cancel for her [going out or meeting people] … there are some places you can't go because of her [too crowded]”
	21: “Currently I don't go out, I don't meet anyone … and even if I go out we go out together [participant and child]”
Financial impact	
Without financial support we could not care for our child	Government:
	11: “thanks to Allah I wasn't affected financially [in relation to healthcare] because it is a government hospital”
	23: “May Allah bless them [the government], the government is paying for all the care”
	Nonprofit organization:
	12: “We receive financial support from the [nonprofit organization]” [For healthcare and daily expenses]
	22: “Thanks to Allah the [nonprofit organization] is supporting us financially so we are doing fine”
We were struggling financially	05: “I was struggling … sometimes my son would drive us around and sometimes we would take an “Uber” … you know “Uber” is very costly!”
	08: “Look at us [parents]! our lives are unstable! My husband doesn't have a job, we are not doing well financially!”
	15: “The care is expensive! Before my husband could afford it, you know the physical therapy was in a private center … but now he [husband] has financial problems”

### Psychological impact

#### Shock and disbelief

Participants expressed a range of emotions as they described the overwhelming upheaval that was ongoing in their lives due to the dramatic altering in their child's health. Mothers and caregivers expressed similar responses of “shock and disbelief” when informed about their child's diagnosis and prognosis. The mothers and caregivers “couldn't believe” it that their lives and those of their children had changed and would never be the same. An exemplar that is representative of the mother's responses of shock and disbelief are reflected in the following participants' quotes:

“It was a huge shock to me. It was a shock to me” (02).

“I was in shock at first, cancer!” (12).

Another participant expressed her disbelief by saying:

“At the beginning when they told me I couldn't believe the doctors. The doctors at [hospital in Jeddah] did the blood works again and I couldn't believe it! I couldn't believe that she has this disease [leukemia]” (22).

#### My heart was breaking

Nearly half of the mothers discussed the heartbreak that they were experiencing at the pending loss of their child who was receiving EOLC. Mothers cried as they expressed how their heart was breaking, with one even stating that their heart was “coming out of my chest” as they watched the deterioration of their child's health. In this example, the participant pounded her chest as she responded to the interviewer's question, and shared the following:

“I told my husband that I feel my heart my heart my heart my heart my heart was coming out of my chest!… my heart wasn't in its place” (07).

There were mothers who reported that they had *suicidal ideations* as they could not handle the fact that their child is severely ill. An issue that was raised was that the mothers would rather be the ones to suffer and die instead of their child. However, the mothers explained that the thought of their child suffering more if they were not there to care for them stopped the participants from attempting suicide. The concerns of the mothers were most saliently reflected by the following quote:

“The doctors said that the biopsy they got from the lymph nodes showed that he has severe cancer in his lymph nodes. I wasn't doing well, psychologically! I thought of ending my life! But then I thought who is going to take care of my son if I'm not there?!” (21).

#### How could I let go of a piece of my heart?

Most of the participants described being very *attached* to their children. A special bond had formed between them and their caregivers. The strong attachment to each other was discussed by one caregiver as:

“Even when I go home for a break I can't stay long … I can't wait for the day to end so I can come back to hospital…. when I come back she gets better…. I don't feel obligated … it's just my heart is so attached to her. No matter how long I spend time away from her, she is always on my mind” (13).

Being with their dying child was a source of *strength* for participants, which kept them surviving all this time. Strength was the participants' way of showing their love for the child. A mother shared her experience by stating:

“I remember when I used to cry my son was young, he used to say why mama cry? Why mama cry? I used to tell him I have a headache, then he says ok mama no cry … so I made myself stronger … I will never show my weakness! I don't want my child to see it and get sick…. I have to make myself strong … even if I'm shattered from the inside, the outside has to be strength” (12).

### Physical impact

#### When you are sad, you don't eat well

A majority of the caregivers shared that having a child with complex health care needs, especially at EOL, was exhausting. Their participant's exemplars of the physical impact of caring for their child at EOL focused on their lack of appetite and sleep disturbances. Changes in their appetite were evidenced by participants sharing that they had lost *their appetite* and experienced a great deal of *weight loss*. Eating well was not a priority for the respondents, as they expressed their sadness over the child, as evidenced by the following:

“When you are sad you don't eat well, I mean I don't want to eat” (10).

Mothers provided descriptions of their alterations in dietary intake, with the decreased intake resulting in subsequent weight loss, as evidenced by one of the more representative quotes:

“Yeees I lost weight, I mean I used to be 86 kilos [189 pounds] and now I am 74 [163 pounds]. I wasn't eating! I mean I wasn't eating, and my body is not the type that lose weight fast!” (07).

#### I sleep, but not sleep

*Sleep deprivation* was ongoing due to the complexity of their child's health care requirements. In addition, the participants discussed being overwhelmed and worrying about the welfare of their child. The caregivers wanted to confirm that the child's needs were met so the child could sleep well, stating:

“No sleep … I stay awake two days like this. Sometimes three days. And if I sleep, I sleep half an hour, not an hour … because I have to take care of my son, his milk, change his diapers, suction his sputum. Next to him like this I could sleep [mother laying in bed with the child]” (02).

### Social impact

#### Isolated themselves from the entire world

The caregivers shared that they *isolated themselves* from everyone around them, because socializing with others was not their priority, as evidenced by the following:

“I didn't feel like going out or meeting anyone … I was kind of isolated during that time, I didn't even talk to my family! isolated” (18).

Also, respondents shared that they were constantly distracted thinking of their child, which made socialization insignificant. A caregiver shared her challenges socializing by stating:

“Honestly, I was sitting with people, but I don't feel them. All the time, I was thinking of her [child]” (03).

#### Can't go out and leave the child alone

A majority of the participants remained at their child's side the entire time. When the child was admitted to the pediatric or neonatal intensive care units, respondents reported not being allowed to stay with their child. Nevertheless, caregivers agonized about *leaving the child alone* at the hospital. The participants worried that the child's health might deteriorate while they were away, as a mother reported:

“I can't go out, I can't leave my son alone … I worry my son gets sick and I'm not around … I can't go anywhere” (15).

### Financial impact

#### Without financial support we could not care for our child

Most of the participants (*n* = 17) were recruited from urban hospitals in Jeddah, Saudi Arabia. The participants recruited from hospitals discussed at great length the care that their child had and the *financial support they were receiving from the Saudi Arabian government*. A respondent shared:

“It is a government hospital … but in general there are no expenses because it is a government hospital, they don't ask you for anything … the government is paying all the hospital expenses … the government is providing the best care in the hospital” (01).

The remaining participants (*n* = 7) were recruited from the community. The respondents shared that they were receiving *financial support from a nonprofit organization*, which provided financial support for the child and the family. The caregivers highly appreciated the support, as evidenced by the following:

“Also the [nonprofit organization] are very supportive. Without them we could not be able to care for our daughter” (18).

#### We were struggling financially

The ongoing nature of the child's health needs led to inevitable *financial challenges*. Some respondents shared that their child was admitted to a private hospital before the cost or care required them to be transferred to a government hospital. Hence, the families were struggling paying for their child's health care. A caregiver stated:

“I mean we spent over 70 thousand [Saudi Riyals = 18,666 Dollars] paying hospitals for appointments and MRIs I mean a loooot … EEG, follow up appointments and other things … can you believe this! We spent one night at [private hospital] we had to pay around 15 thousand [Saudi Arabia = 4,000 Dollars]!” (07).

Daily expenses at home needed to be maintained, even though the child was sick in the hospital, as a mother stated:

“His father's salary is not much, we can barely afford transportation, school necessities and the kitchen, you know we have to buy food every other day, my son gets hungry” (12).

## Discussion

In this study, the primary caregivers were overwhelmingly mothers, who reported being in shock and disbelief when told about their child's prognosis, as their life was flipped upside down. Similarities to the shock and disbelief of the mothers in this study was reported in the Young study,^8^ which explored the stressors of parents whose child was diagnosed with cancer. The researcher found that there were common stressors experienced by parents of children diagnosed with cancer, including shock on receiving the diagnosis.

Unlike the Young study,^8^ mothers in our study were not currently experiencing shock about the specific diagnosis, but the life-limiting aspect of their child's illness. The differences between the experiences of parental shock in the two studies were that the mothers in the current study had received their child's diagnosis long before the EOL prognosis. Two different points of illness (diagnosis vs. EOL) are being compared between this study and the Young investigation. However, the similarity is that when parents learn of their child's cancer diagnosis, one of their first concerns is that their child is dying,^9^ which is the same worry of the mothers in this study, that their child is dying.

Mothers in the current study reported being heartbroken when they saw that their child was in pain and suffering. Suffering was also found to be a concern in a study by Arabiat and Altamimi,^10^ where the investigators explored the beliefs of Jordanian mothers regarding their children's cancer. The Jordanian mothers shared that seeing their child physically suffering was painful and heartbreaking. Expressing that their heart was breaking was a common theme across both the Arabiat and Altamimi,^10^ investigation and the current study. Respondents expressed that not being able to relieve their child's suffering led to their heartbreaking. Of great concern was that a couple of mothers in the current study reported having experienced intermittent suicidal thoughts.

Thoughts of suicide were not the focus of the current study, nor was it the aim of a study conducted by Zetumer et al.^11^ In the investigation by Zetumer et al,^11^ the researchers examined parental behaviors and thoughts after the death of a child. It is important to note that although the investigators in the Zetumer et al,^11^ study were focused on grief, the parents shared that their grief was so intense that suicidal thoughts came repeatedly to their minds.

Although only a couple of mothers in the current study expressed thoughts of suicide, the grief of participants in the Zetumer et al^11^ investigation reached the point of many parents considering engaging in suicidal behaviors or attempting suicide. However, mothers in the current study who also contemplated suicide shared that their worries about who would care for their child if they were gone prevented them from taking any action.

The prolonger hospitalization of their child, due to their deteriorating health at EOL, was a contributing factor to their alterations in appetite and sleep habits. The mother's views on what impacted their physical health in the current study is similar to other primary caregiver's physical fatigue and exhaustion when caring for any child with complex health care needs.^12,13^ Worry about the child's deteriorating health and the potential loss of life led mothers in this study to not eat, and their sleep was disturbed by the machines that monitored their child's health status while in the hospital.

Alterations in dietary patterns during prolonged hospitalization were found to be an issue in a study by Church et al^12^ In the investigation by Church et al,^12^ parents were found to react with varying responses to the prolonged hospitalization of their children. Some parents reported losing their appetite and experiencing subsequent weight loss, whereas other parents responded to the prolonged hospitalizations by taking in excess calories and expressed frustration over their weight gain.

Parental responses to having a child with a significant health condition is not limited to altered dietary patterns, but, according to Kish et al,^13^ also leads to altered sleep patterns. In the study by Kish et al,^13^ sleep deficits were reported by the mothers who were caring for their child with a chronic condition. Mothers in the Kish et al investigation, as in the current study, appeared to focus on caring for the child's needs, to the detriment of their own well-being.

Previous studies have shown that mothers isolated themselves to provide their child EOLC.^14,15^ Broady,^16^ a literature review was conducted on the experiences of caregivers providing EOLC. The caregivers dedicated their time to the patient and neglected themselves. Like the current study, the respondents spent all their time with the child, whether at home or the hospital.

Globally, it is known that EOLC cost is considered a burden to families and health care systems.^17–19^ Rowland et al^20^ explored the expenditure of families providing cancer EOLC at home. Participants reported that their most frequent expenditures were for transportation, meals, medical care supplies, and household bills. In the current study, the participants also mentioned struggling financially due to increased expenditures since their child started receiving EOLC.

One of the benefits in Saudi Arabia is that the citizens of the country receive free public health care service.^21^ Al-Hanawi et al^21^ conducted a study that examined the views of Saudi Arabians on the receipt of public health care. The investigators found that overwhelmingly, the citizens in Saudi Arabia were very appreciative of the Saudi Arabian government providing them with health care services. Without government support, most primary caregivers in this study would not have the resources to pay for the child's EOLC.

It is important to note that a subset of participants in this study (29%) had children who were being cared for in private hospitals. This subset of mothers shared that a nonprofit organization provided financial support for their families, because their child had been diagnosed with cancer. The organization covered their child's expenses in the private hospital, their medications, and housing for families who were not residents within the local community. Non-profit foundations, similar to the one discussed in this study, for example, HealthWell Foundation,^22^ were found to provide financial support to underinsured patients with prescription co-pays, out-of-pocket expenses, and pediatric treatment costs.

The findings of the study provided insight into the experiences of Saudi Arabian primary caregivers caring for their children at EOL; however, the study had limitations. Given the small sample size (*N* = 24), this study cannot be generalized to a larger population. However, given that this study focused on the personal experiences of mothers caring for their children at EOL, generalizability was not the aim of this study. The study focused on the experiences of female caregivers instead of male or extended family caregivers, and future studies should address the broader range of familial perspectives.

A potential limitation of this study was obtaining the purposive sample (29%) of participants through snowball sampling, as it can prevent the findings from being generalized to a broader population. As phenomenology is specifically designed to enable a greater depth of understanding regarding a specific phenomenon, the emphasis with recruitment is on ensuring the participants meet the inclusion criteria. Therefore, snowball sampling was one of the appropriate ways to recruit participants for this study.

Given the findings from this study, there are several implications for health care professionals to consider when children are receiving EOLC. The mothers in this study clearly identified that having a child who is receiving EOLC affects the whole family, including parents, children, and extended family members. Implementing family-centered care must be considered, as the family is integral to the well-being of the child at EOL. It is hard for a mother to provide care for her child at EOL, if they are overwhelmed with ensuring their other children are cared for.

Ensuring the needs of the family are met allows the family to then focus on the well-being of the child receiving EOLC. One way to accomplish this is to have private rooms that allow family members to visit throughout the day, regardless of age. Respite services for the siblings at home would enable mothers to focus on caring for the child receiving EOLC.

Respite care for the mothers is another consideration. Have a play or music therapist scheduled to work with the child at EOL at set times, so that mothers can relax, eat undisturbed, or take care of the family at home. Alternatively, ensuring the hospital room is set up to meet the needs of mothers as well as their child at EOL is important. A bed in the room for sleeping next to the child, an enclosed shower, TV for distraction, and having meals delivered would allow mothers to focus on caring for their child at EOL.

Resources are essential to support the mother's provision of emotional care at the bedside of the child at EOL. Essential resources can vary, depending on the mother. If the mother does not have the resources to travel to the hospital, then transportation, or financial support for travel may be required. Communication is a resource that is also important to consider. Having electronic devices that allow the mother to monitor what is happening at home and stay in touch with family members would be beneficial to strengthening the family unit.

## Conclusion

Gaining a greater understanding of the needs of the child at EOL and their families can assist care providers in ensuring they receive the highest quality of care. This descriptive phenomenological study sheds light on the primary caregivers' experiences caring for a child at EOL in Saudi Arabia. Through individual interviews, the investigator identified the holistic impact on primary caregivers caring for a child at EOL. Similar to what has been reported in the literature, primary caregivers were affected psychologically, physically, socially, and financially while caring for a child at EOL. The findings of this study can be used to and contribute to the body of knowledge on EOLC in Saudi Arabia and globally to improve the care of children at EOL.

## Abbreviations Used

EOL: end of life
EOLC: end of life care
